# Anxiety disorders across middle childhood and early adolescence in a UK population‐based cohort

**DOI:** 10.1002/jcv2.12089

**Published:** 2022-06-24

**Authors:** Isabel Morales‐Muñoz, Danielle Hett, Clara Humpston, Pavan K. Mallikarjun, Steven Marwaha

**Affiliations:** ^1^ Institute for Mental Health University of Birmingham Birmingham UK; ^2^ Department of Public Health Solutions Mental Health Unit Finnish Institute for Health and Welfare Helsinki Finland; ^3^ National Centre for Mental Health The Barberry, Birmingham and Solihull Mental Health Trust Birmingham UK; ^4^ Department of Psychology University of York York UK; ^5^ Early Intervention Service Birmingham Women's and Children's NHS Trust Birmingham UK

**Keywords:** ALSPAC, anxiety disorders, early adolescence, trajectories

## Abstract

**Background:**

Patterns of development and underlying factors explaining anxiety disorders in children and adolescents are under‐researched, despite their high prevalence, impact and associations with other mental disorders. We aimed to a] understand the pattern and persistence of specific anxiety disorders; b] examine differing trajectories of symptoms of specific anxiety disorders and; c] examine socio‐demographic and health‐related predictors of persistent anxiety disorder‐specific symptoms, across middle childhood to early adolescence.

**Methods:**

The current study used data from 8122 participants in the Avon Longitudinal Study of Parents and Children birth cohort. The Development and Wellbeing Assessment questionnaire was administered to parents to capture child and adolescent anxiety total scores and DAWBA‐derived diagnoses. Separation anxiety, specific phobia, social anxiety, acute stress reaction, and generalized anxiety at 8, 10 and 13 years were selected. Further, we included the following socio‐demographic and health‐related predictors: sex, birth weight, sleep difficulties at 3.5 years, ethnicity, family adversity, maternal age at birth, maternal postnatal anxiety, maternal postnatal depression, maternal bonding, maternal socio‐economic status and maternal education.

**Results:**

Different anxiety disorders presented different prevalence and patterns of development over time. Further, latent class growth analyses yielded a trajectory characterized by individuals with persistent high levels of anxiety across childhood and adolescence; for specific phobia (high = 5.8%; moderate = 20.5%; low = 73.6%), social anxiety (high = 3.4%; moderate = 12.1%; low = 84.5%), acute stress reaction (high = 1.9%; low = 98.1%) and generalized anxiety (high = 5.4%; moderate = 21.7%; low = 72.9%). Finally, the risk factors associated with each of the persistent high levels of anxiety disorders were child sleeping difficulties and postnatal maternal depression and anxiety.

**Conclusions:**

Our findings show that a small group of children and young adolescents continue to suffer from frequent and severe anxiety. When considering treatment strategies for anxiety disorders in this group, children's sleep difficulties and postnatal maternal depression and anxiety need to be assessed as these may predict a more prolonged and severe course of illness.

## Graphical abstract

Through data from a large cohort, this study offers novel findings concerning the trajectories of anxiety disorders and factors associated with persistent anxiety disorders across childhood and adolescence. We found that distinct anxiety disorders presented different prevalence and patterns over time, and we detected persistent high levels of anxiety across childhood and adolescence for specific phobia, social anxiety, acute stress reaction and generalized anxiety. Finally, child sleeping difficulties and postnatal maternal depression and anxiety were the most relevant risk factors for persistent high levels of anxiety disorders.

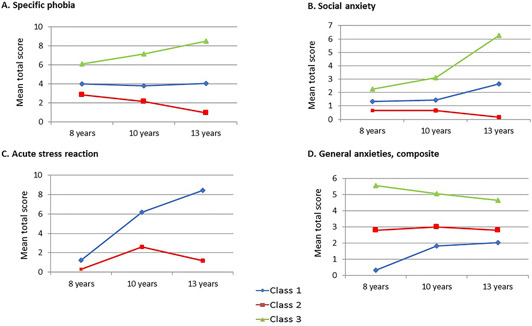

## INTRODUCTION

Anxiety disorders are among the commonest mental disorders in children (Merikangas et al., 2010). Lifetime prevalence is 32%–33% (Bandelow & Michaelis, 2015), with similar rates for children and adolescents (Achenbach et al., 1998; Merikangas et al., 2010). Several anxiety disorders in children and adolescents exist, including separation anxiety, specific phobia, social phobia, or generalized anxiety disorder (GAD) (Copeland et al., 2014). Compared to other mental disorders, anxiety disorders have the earliest age of onset, and are associated with significant impairments in social and academic functioning (Essau, Conradt, & Petermann, 2000). They are highly comorbid with the full range of mental disorders in young people, often represent the first clinical contact and are risk factors for several mental disorders in adulthood (Copeland et al., 2014; Doering et al., 2019). Further studies characterizing anxiety in childhood and adolescence are needed to better understand the impact of anxiety in paediatric populations.

**Key points**

- Patterns of development and underlying factors explaining anxiety disorders in children and adolescents have been an under‐researched area, despite their high prevalence, impact on social and educational functioning and associations with other mental disorders.
- We identified a cohort of young people characterized by persistent high levels of anxiety across childhood and adolescence in the form of specific phobia, social anxiety, acute stress reaction and generalized anxiety.
- Child sleeping difficulties and postnatal maternal depression and anxiety were the most relevant risk factors for persistent high levels of anxiety disorders.
- There is a specific group of children and adolescents who are especially prone to persistent and severe anxiety disorders.

Importantly, the differentiation between normal anxiety symptoms and pathological anxiety disorders can be particularly difficult in children because they manifest many fears and anxieties as part of typical development (Muris et al., 1998). This differentiation between normal anxiety symptoms and pathological disorders can be framed within the dimensional model, where mental illness and positive mental health reflect distinct continua rather than the extreme ends of a single spectrum (Greenspoon & Saklofske, 2001). Further, a strong continuity of anxiety symptoms may evolve to clinically relevant anxiety disorders, thus defining those specific cases at higher risk for persistent anxiety symptomatology, which subsequently would lead to clinical anxiety, is crucial. Studies examining the developmental course of anxiety symptoms at a population level indicate an overall decline in anxiety symptoms from childhood to adolescence (Allan et al., 2014; Olatunji & Cole, 2009), but this trend is likely not specific to all children. These previous studies also report between 2‐to‐5 different anxiety trajectories, and most importantly, they all describe a specific group characterized by persistent high levels of anxiety. However, most of these studies have investigated anxiety as a single dimension, rather than focussing on anxiety disorders, which clinicians would regard as more important. To better understand the specific course of anxiety, trajectories of the different anxiety disorders need to be individually examined, so that we can understand their development over time allowing clinicians to better identify those most at‐risk of severe problems, and target treatment. By focusing on specific anxiety disorders rather than anxiety disorders in general, we aimed to better characterize and understand each of these disorders individually early on life, which potentially would help to design better targeted and individualized early intervention programs in anxiety. However, it is important to highlight that anxiety disorders tend to co‐occur frequently among themselves (Essau et al., 2018), and that many anxiety disorders share common clinical features such as extensive mental anxiety, physiological anxiety symptoms, behavioral disturbances, and associated distress or impairment (Beesdo et al., 2009).

To date, only two studies have examined different trajectories of anxiety disorders over time. One, assessed anxiety trajectories among social phobia, separation anxiety, GAD and panic disorder in 825 adolescents and found between 2‐to‐4 trajectories for social phobia, GAD and panic disorder, demonstrating all of them an elevated symptom trajectory (De la Torre‐Luque et al., 2020). The second study used a larger cohort of adolescents (*N* = 2200) and reported that anxiety disorders evidenced a decrease in symptoms which levelled off and then increased again from mid‐adolescence (GAD, social phobia, separation anxiety) or late‐adolescence (panic disorder) (Van Oort, Greaves‐Lord, Verhulst, Ormel, & Huizink, 2009). These studies are crucial to better understand the heterogeneity in their symptom courses; however, further research is needed, particularly larger sampled population‐cohort studies across middle childhood to early adolescence.

There is also a clinical need to understand the predictors associated with less favourable illness course trajectories (i.e., the *persistence* of anxiety) to aid assessment of prognosis and these remain scarce. Previous research suggests that temperament, internalising problems and being bullied are risk factors for persistent anxiety in adolescence (Van Oort, Greaves‐Lord, Ormel, Verhulst, & Huizink, 2011). However, the socio‐demographic and health‐related factors associated with persistent anxiety across childhood and adolescence remain unclear. These factors might be particularly relevant for clinicians as these might be more modifiable (i.e., more treatable conditions which can be targeted as a prevention against anxiety disorders), and thus easier to treat. Further, it is still unknown whether these factors associate similarly or distinctively with each of the anxiety disorders.

This study will address important gaps in our knowledge about anxiety in children and adolescents. The study aims were to: (a) understand the pattern and persistence of specific anxiety disorders; (b) examine differing trajectories of symptoms of specific anxiety disorders; and (c) investigate socio‐demographic and health‐related associations of those trajectories, across middle childhood and early adolescence (i.e., 8–13 years).

## METHOD

### Participants

The Avon Longitudinal Study of Parents and Children (ALSPAC) is a UK birth cohort study examining the determinants of development, health and disease during childhood and beyond (Boyd et al., 2013; Fraser et al., 2013). Pregnant women resident in Avon, UK with expected dates of delivery 1st April 1991 to 31st December 1992 were invited to take part in the study. The ALSPAC study website contains details of all the data available (http://www.bristol.ac.uk/alspac/researchers/our‐data/). The initial number of pregnancies enrolled was 14,541. For this study, we included all participants with information available at 8 (*N* = 8122), 10 (*N* = 7658) and 13 years old (*N* = 6906). Further details of this cohort are described in the Supporting Information. Ethical approval was obtained from the ALSPAC Law and Ethics Committee and local research ethics committees. Informed consent was obtained from participants following the recommendations of the ALSPAC Ethics and Law Committee.

### Outcomes

The Development and Wellbeing Assessment (DAWBA) (Goodman et al., 2000) was administered as parent‐report questionnaire to capture child and adolescent psychopathology corresponding with the International Classification of Diseases–10th Revision (ICD‐10) and Diagnostic and Statistical Manual of Mental Disorders—4th Edition (Diagnostic and Statistical Manual of Mental Disorders—4th Edition (DSM‐IV)) criteria. Further details of the DAWBA are provided in the Supporting Information. To investigate the most prevalent and clinically relevant anxiety disorders in childhood and adolescence, we focused on the following DAWBA anxiety classification at 8, 10 and 13 years: separation anxiety, specific phobia, social anxiety, acute stress reaction, and generalized anxiety. A computerized algorithm was used to derive ordered categorical variables (DAWBA bands, with 6 categories) based on the ICD‐10 and DSM‐IV criteria (Goodman et al., 2000). The top two levels of the DAWBA bands were used as DAWBA‐derived diagnoses. This follows previous studies using the DAWBA bands, where the top two levels were used as computer‐generated DAWBA diagnoses, and these DAWBA bands were validated in 7912 British children (7–19 years) and 1364 Norwegian children (11–13 years), using clinician‐rated DAWBA diagnoses as a gold standard (Goodman et al., 2011). Briefly, the top two DAWBA bands were compared with the ‘gold standard’ clinician‐rated diagnoses, and the computer‐generated diagnoses yielded very similar results regarding associations with risk factors. This suggests that the DAWBA bands function well as ordered categorical measures, show a dose‐response association with mental health service contact and can also be used to examine dose‐response associations with risk factors.

Also, we calculated the total scores for each anxiety disorder to obtain anxiety trajectories. Separation anxiety total score was calculated using the separation anxiety subscale, specific phobia total score using the particular fears subscale, social anxiety total score using the social fears subscale, and acute stress reaction total score using the stress reaction subscale. Further, we created a composite measure of generalized anxiety, by calculating the arithmetic mean of: (i) generalized anxieties total score, which was calculated using the generalized anxieties subscale; and (ii) generalized anxieties symptoms score, which was calculated using the generalized anxieties symptoms subscale. We used this composite score for the purpose of our study to allow capture of two dimensions of anxiety (cognitive dimension, from the generalized anxieties total score; and symptomatology dimension, from the generalized anxieties symptoms score), rather than only one. For each of the items, the response alternatives provided to the parents where 1 = Yes; 0 = No. A description of each of the items comprising each of the DABWA anxiety total scores is provided in the Supporting Information (Table S1).

### Predictors

Multiple family risk factors were assessed using the Family Adversity Index (FAI) during pregnancy, at 2 years, and at 4 years. Points were summed at each time point for a total FAI score across the 3 time points.

Maternal postnatal depression at 8‐week after birth was measured with the Edinburgh Postnatal Depression Scale (Cox et al., 1987). The total score was used for this study.

Maternal postnatal anxiety at 8‐week after birth was measured using the Crown‐Crisp Index (Birtchnell et al., 1988). For this study, only the total score from the anxiety subscale was used.

Maternal bonding score at 8 months was obtained from combining the two following subscale scores: maternal enjoyment and maternal confidence (Ellis et al., 2020).

Prenatal maternal socioeconomic status was measured using the Cambridge Social Interaction and Stratification Scale, which provides a total score (Prandy & Lambert, 2003).

Prenatal maternal education was measured by asking the highest qualification achieved. This was coded into: no qualifications, Certificate of Secondary Education or General Certificate of Secondary Education, O level or equivalent, A level or equivalent, teaching or nursing qualification, and university degree. A dichotomous variable was created: O level or lower = 0, A level or higher = 1.

Children sleep difficulties total score at 3.5 years was created by summing the following variables: child refused to go to bed, woke very early, problems going to sleep, nightmares, got up after being put to bed, woke at night, and got up after few hours of sleep.

Finally, child's sex, birth weight and ethnicity (white vs. non‐white), and maternal age when baby was born were also included.

All these variables were included as predictors based on previous evidence supporting the relevant role of each of them on subsequent mental health problems, including anxiety. For instance, previous evidence supports that early life adversities, including family adversities (Draisey et al., 2020), maternal postnatal depression (Morales‐Munoz et al., 2022) and anxiety (O’Connor, Heron, Golding, & Glover, 2003), early bonding (Winston & Chicot, 2016), socio‐economic status (Reiss et al., 2019) parents education (Torvik et al., 2020), and early sleep difficulties (Morales‐Muñoz et al., 2020), among others, are all implicated in the development and maintenance of childhood anxiety disorders. In addition, women (McLean et al., 2011), individuals from socially disadvantaged racial and ethnic populations (Williams, 2018), and children who are born with lower weight (Nomura et al., 2007) and whose mothers are older (Tearne et al., 2016) are all at higher risk for developing future anxiety problems.

Further details of each of the predictors, including a description of the variables/subscales are provided in the Supporting Information (Table S2).

### Statistical analysis

A multi‐staged analysis plan was developed. The first stage was conducted in SPSS‐v25 to describe the normative patterns of specific DAWBA‐derived anxiety diagnoses and symptoms across childhood and adolescence, using descriptive analyses.

A latent class growth analyses (LCGA) was then conducted using Mplus‐v8 to investigate differing trajectories of symptoms of specific anxiety disorders across childhood and adolescence. The indicator variables were DAWBA anxiety total scores at 8, 10 and 13 years. We ran LCGA for each DAWBA anxiety disorder (i.e., separation anxiety, specific phobia, social anxiety, acute stress reaction, and generalized anxiety). Several models were fitted by increasing the number of classes (Jung & Wickrama, 2008). The best fitting classification model was initially chosen according to fit indices (i.e., Bayesian Information Criteria [BIC] and Vuong‐Lo‐Mendell‐Rubin [VLMR] test), and entropy. Finally, to decide the optimal class solution, an emphasis was placed on large enough group sizes. Missing values due to attrition were handled by the Full Information Maximum Likelihood estimation method.

Thirdly, SPSS‐v25 was used to define socio‐demographic and health‐related risk factors predicting persistent high levels of DAWBA anxiety disorder‐specific symptoms. We conducted separate logistic regressions, for each DAWBA anxiety disorder. The explanatory variables were: (i) child‐related predictors were sex, birth weight, sleep difficulties at 3.5 years, ethnicity, and family adversity; and (ii) mother‐related predictors were age at birth, postnatal anxiety, postnatal depression, bonding, socio‐economic status and education. For the outcome, we created a new dichotomous variable, based on the best model fit classes obtained from the LCGA, and this was done for each DAWBA anxiety disorder: the class representing persistent high anxiety was recoded as 1, while the other class/es was/were recoded as 0.

In relation to attrition rates in our study, 48.1% of the original sample was lost to attrition by the 8‐year follow‐up, 50.3% by the 10‐year follow‐up, and 55.9% by the 13‐year follow‐up. Therefore, and to deal with missingness, we conducted logistic regressions to identify significant factors associated with attrition. The individuals associated with attrition in adolescence had younger mothers, shorter gestational age, higher family adversity problems, weighed less at birth and were more frequently non‐white (Table S3 in the Supporting Information). Using the variables associated with selective dropout as predictors, we fitted a logistic regression model to determine weights for each individual using the inverse probability of response. We used this weighting variable in the logistic regression analyses. We decided against the use of multiple imputation as data in ALSPAC is unlikely to be missing at random.

## RESULTS

### Development of anxiety disorders

**FIGURE 1:**
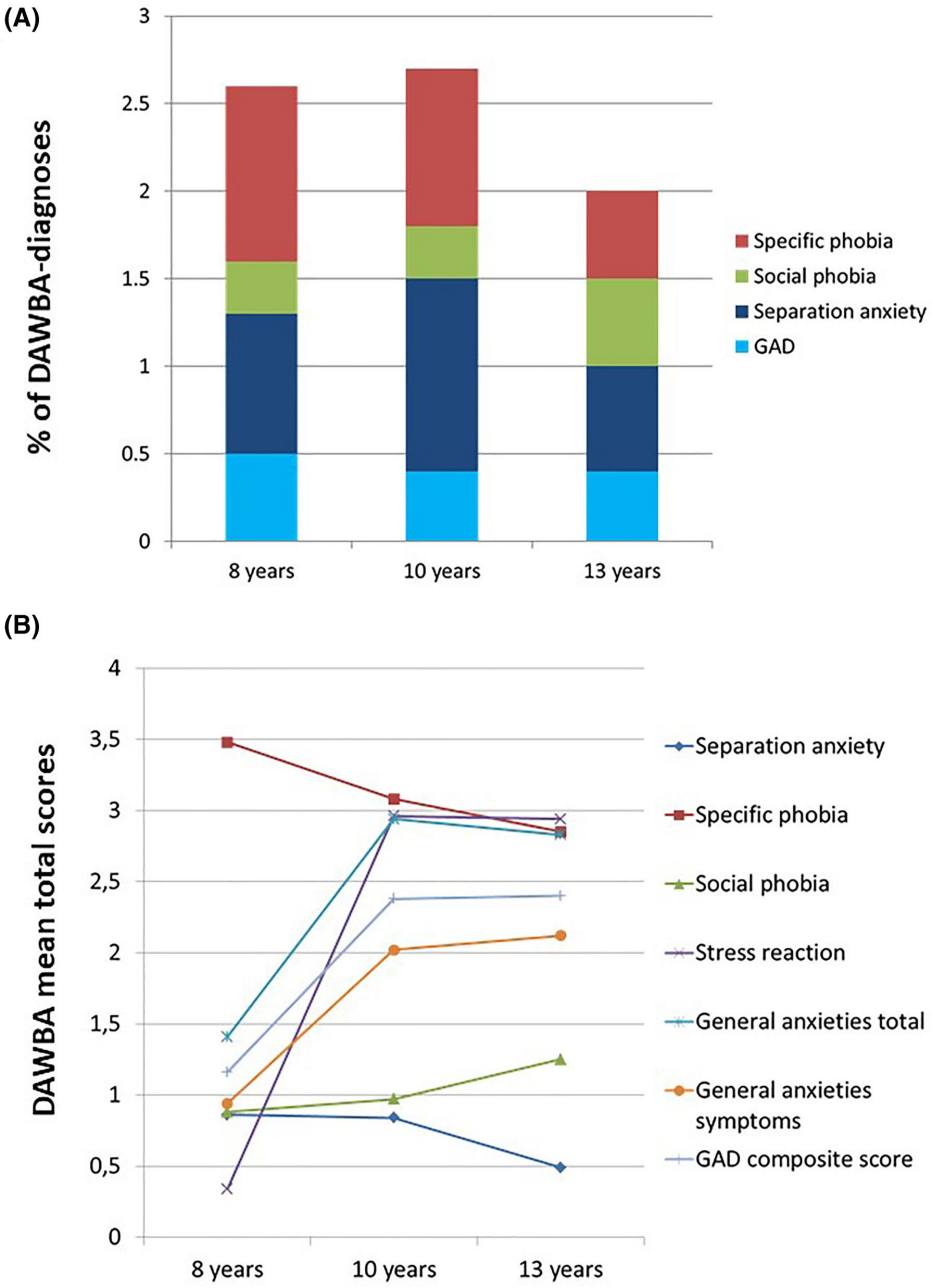
Prevalence and developmental course of Development and Wellbeing Assessment (DAWBA) anxiety disorders from 8 to 13 years old, for anxiety diagnosis and anxiety symptomatology. (A) Represents the prevalence for the diagnosis for each anxiety disorder, at 8, 10 and 13 years. Purple color represents specific phobia, green social phobia, red separation anxiety and blue generalized anxiety disorder (GAD). The graph indicates that at 8 years, specific phobia is the most prevalent diagnosis, while at 10 years the most prevalent is separation anxiety. At 13 years, separation anxiety seems to be still the most prevalent diagnosis. Concerning social phobia, the prevalence of this diagnosis is the lowest at 8 and 10 years, while it considerably increases at 13 years to become the second most prevalent one. Finally, concerning GAD, this is the second lowest at 8 years, while the one with lowest prevalence at 10 and 13 years. (B) Reflects the mean total scores for the symptomatology for each anxiety disorder, at each time point. Specific phobia shows a decreasing pattern over time with average mean values around 3. Generalized anxiety shows an increasing pattern over time, specially from 8 to 10 years, with average mean values at 10 and 13 years being close to 3. Social phobia presents overall low mean values over time, with the mean scores showing an increasing trend over time (i.e. mean close to 1 at 8 and 10 years, and close to 1.5 at 13 years); and separation anxiety shows a decreasing pattern over time, with mean values being lower than 1 at all the time points. Finally, acute stress reaction shows an increasing pattern over time, especially from 8 to 10 years old, with the mean values increasing from 0.2 at 8 years, to 3 at 10 and 13 years old

Concerning DAWBA‐derived anxiety diagnoses, different anxiety disorders presented different patterns of development and courses over time (see Figure 1A). Further, concerning the total prevalence of all the anxiety disorders at each time point, including the presence of at least one of the assessed disorders, we found the highest prevalence at 10 years (2.7%), then at 8 years (2.6%) and finally at 13 years (2%).

Specific phobia was the most prevalent anxiety diagnosis at 8 and 10 years. Separation anxiety was the second most prevalent at 8 years, while it was the most prevalent at 10 and 13 years. Generalized anxiety disorder was the third most prevalent at 8 and 10 years, and the less prevalent at 13 years. Finally, social phobia was the less prevalent at 8 and 10 years, while the second most prevalent at 13 years, together with specific phobia. Interestingly, the prevalence rates for each anxiety disorder seem very similar at age 13, and also there is a clear variation in the course for each specific disorder (e.g., separation anxiety and specific phobia peak at 10 years, while social anxiety is more prevalent at 13 years). See Table 1 for a description of the prevalence for each DABWA‐derived diagnosis.

**TABLE 1:** Descriptive values of sociodemographic and health‐related variables, and anxiety measures

	8 years old		10 years old		13 years old	
Social and health risk factors	N	%	N	%	N	%
Sex (boys/girls)	4235/4007	51.4/48.6	3938/3869	50.4/49.6	3462/3444	50.1/49.9
Ethnicity (white/non‐whites)	7808/145	98.2/1.8	7106/125	98.3/1.7	6323/104	98.4/1.6
Maternal education, pregnancy (O levels or lower/a levels or higher)	3911/3309	54.2/45.8	3477/3066	53.1/46.9	3010/2835	51.5/48.5

	Mean	SD	Mean	SD	Mean	SD
Birth weight, kg	3.42	0.55	3.42	0.54	3.43	0.54
FAI, total score	4.06	4.10	3.93	4.01	3.86	4.01
Maternal postnatal depression, total	5.86	4.61	5.82	4.63	5.78	4.61
Maternal postnatal anxiety, total	3.54	3.27	3.51	3.26	3.50	3.27
Maternal socio‐economic status, total	54.59	13.47	54.93	13.54	55.25	13.45

**DAWBA**	Yes (%)	No (%)	Yes (%)	No (%)	Yes (%)	No (%)
DAWBA anxiety diagnoses						
Separation anxiety	69 (0.8%)	8167 (99.2%)	83 (1.1%)	7429 (98.9%)	38 (0.6%)	6463 (99.4%)
Specific phobia	86 (1%)	8150 (99.0%)	67 (0.9%)	7705 (99.1%)	36 (0.5%)	7058 (99.5%)
Social phobia	22 (0.3%)	8214 (99.7%)	26 (0.3%)	7755 (99.7%)	32 (0.5%)	7033 (99.5%)
GAD	41 (0.5%)	8195 (99.5)	33 (0.4%)	7724 (99.6%)	31 (0.4%)	7015 (99.6%)

	Mean	SD	Mean	SD	Mean	SD
DAWBA anxiety total score^a^						
Separation anxiety	0.86	1.98	0.84	2.04	0.49	1.46
Specific phobia	3.48	2.51	3.08	2.76	2.85	2.59
Social anxiety	0.88	1.66	0.97	1.70	1.25	1.91
Acute stress reaction	0.34	1.52	2.96	3.87	2.94	3.81
Generalized anxieties, composite score	1.16	1.59	2.37	1.61	2.40	1.71

Abbreviations: DAWBA, The Development and Well‐Being Assessment; FAI, Family Adversity Index; GAD, Generalized Anxiety Disorder.

^a^ Maximum and minimum scores for DAWBA anxiety total score: Separation anxiety (0–20); Specific phobia (0–12); Social anxiety (0–19); Acute stress reaction (0–22); Generalized anxieties, composite score (0–13).

Concerning the total scores of DAWBA anxiety symptoms, we also found different patterns of development across time points (see Figure 1B). Specific phobia showed a decreasing pattern over time with average mean values around 3 at each time point. Generalized anxiety showed an increasing pattern over time, specially from 8 to 10 years, with average mean values at 10 and 13 years being close to 3. Social phobia presented overall low mean values over time, with the mean scores showing an increasing trend over time (i.e. mean close to 1 at 8 and 10 years, and close to 1.5 at 13 years); and separation anxiety showed a decreasing pattern over time, with mean values being lower than 1 at all time points. Finally, acute stress reaction showed an increasing pattern over time, especially from 8 to 10 years old, with the mean values increasing from 0.2 at 8 years, to 3 at 10 and 13 years old. The descriptive values for each of the total scores appear in Table 1.

### Latent classes of anxiety disorders

**FIGURE 2:**
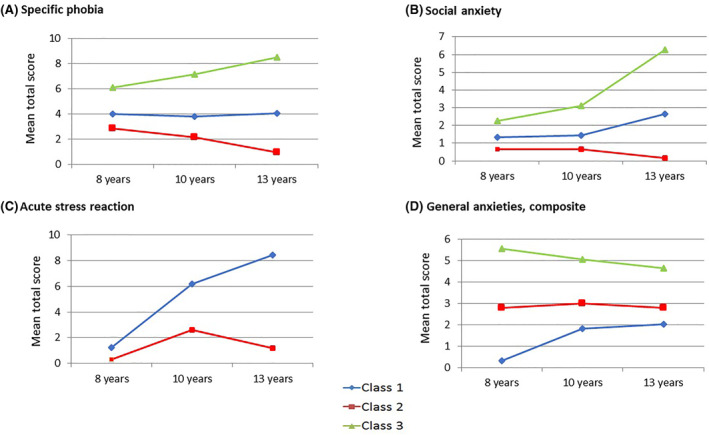
Growth trajectories of specific phobia, social anxiety, acute stress reaction and generalized anxiety across childhood and adolescence. The latent class growth analyses (LCGA) detected a best model fit for 3 classes for all anxiety disorders, except for acute stress reaction with a best model fit for 2 classes. *X* axis represents the three time points in childhood and adolescence, and the *Y* axis represents the mean total score of Development and Wellbeing Assessment (DAWBA) anxiety disorders. (A) reflects the trajectories for specific phobia. Class 3 (green line) is characterized by persistent (and increasing) high levels. Class 1 (blue line) represents persistent (and stable) moderate levels. Finally, class 2 (red line) reflects persistent (and decreasing) low levels. (B) shows the trajectories for social anxiety. These trajectories show a class 3 (green line) characterized by persistent (and increasing) high levels; a class 1 (blue line) reflecting persistent (and increasing) moderate levels; and finally a class 2 (red line) representing persistent (and decreasing) low levels. (C) shows the trajectories for acute stress reaction. These trajectories show a class 1 (blue line) characterized by persistent (and increasing) high levels; and a class 2 (red line) characterized by persistent (and increasing and decreasing) low levels. Finally, (D) reflects the trajectories for the composite score of generalized anxieties. The trajectories show a class 3 (green line) characterized by persistent (and decreasing) high levels; and class 2 (red line) characterized by persistent (and increasing and decreasing) moderate levels; and a class 1 (blue line) characterized by persistent (and increasing) low levels

In the LCGA, we focused on the class for each disorder that related to persistent/high levels, and our LCGA investigated trajectories of ‘symptoms’ of specific anxiety disorders. Table 2 shows the values of log‐likelihood (VLMR, BIC, and entropy) for all models assessed (2‐6 classes) for all anxiety disorders. Overall, a 3‐class model offered the best fit for all, except for acute stress reaction, where a 2‐class model offered the best fit, and for separation anxiety, where no good fit was observed. The trajectories for each anxiety disorder appear in Figure 2.

**TABLE 2:** Bayesian Information Criterion, Vuong‐Lo‐Mendell‐Rubin Likelihood Test *p* Values and Entropy for classes 2–6, for each of the Development and Wellbeing Assessment (DAWBA) dimensions total scores, for the whole sample

	BIC	VLMR‐P	Entropy
Specific phobia			
2 classes	166,936.485	<0.001	0.915
3 classes	161,155.739	<0.001	0.946
4 classes	157,223.012	0.0003	0.964
5 classes	153,379.909	<0.001	0.985
6 classes	150,390.709	<0.001	0.992
Social anxiety			
2 classes	122,303.882	<0.001	0.973
3 classes	113,688.994	<0.001	0.996
4 classes	110,837.446	<0.001	0.986
5 classes	103,768.038	<0.001	0.990
6 classes	102,898.299	0.5053	0.779
Acute stress reaction			
2 classes	115,711.489	0.0079	1.000
3 classes	104,614.972	0.0464	1.000
4 classes	102,645.272	0.4134	0.988
5 classes	93,387.673	0.5974	0.989
6 classes	86,635.657	0.2961	0.990
Separation anxiety			
2 classes	115,984.890	0.0885	0.998
3 classes	106,031.829	0.4123	0.999
4 classes	100,770.832	0.5256	0.994
5 classes	93,356.002	0.3340	0.995
6 classes	91,858.730	0.4925	0.994
Composite score of generalized anxieties			
2 classes	47,499.749	<0.001	0.853
3 classes	45,831.340	0.0014	0.864
4 classes	45,640.278	0.0007	0.817
5 classes	43,290.281	0.0481	0.884
6 classes	43,317.489	0.2610	0.833

Abbreviations: BIC, Bayesian information criterion; VLMR‐P, Vuong‐Lo‐Mendell‐Rubin likelihood ratio test.

Concerning specific phobia, class 3 represented persistent and increasing high levels of anxiety and comprised 5.8% of the sample. For social anxiety, class 3, which reflected individuals with persistent and increasing high levels, comprised 3.4% of the sample. Regarding acute stress reaction, class 2, which defined those individuals with persistent and increasing high levels, comprised 1.9% of the sample. Finally, regarding generalized anxiety, class 3, which represented persistent and decreasing high levels, comprised 5.4% of the sample. The prevalence of cases per class, for the best model fit of each DAWBA anxiety disorder appears in the Supporting Information, Table S4.

Additionally, and following previous research reporting gender differences in anxiety trajectories (Ohannessian et al., 2017), we tested LCGA separately for boys and girls. Latent class growth analyses showed trajectories with good model fit in both genders for specific phobia, social anxiety and generalized anxiety. In all three, a similar trend was observed for boys and girls and we therefore focus on the combined sample in reporting. See Table S5 and figures S1a and S1b in the Supporting Information for further details.

Finally, and given the potential high levels of comorbidity between different anxiety disorders, we additionally tested for the potential significant overlap among the participants who were in the highest‐severity classes for each DAWBA‐derived anxiety disorder. Overall, we observed that the prevalence of cases with overlapping persistent levels of anxiety disorders was relatively low (ranging from 7.7% to 18%), but the statistical values were significant (*p* < .001). See Table S6 in the Supporting Information for all prevalence and statistical values.

### Risk factors and persistent high levels of anxiety disorders

The regression models of our study examined predictors of persistent high levels of disorder‐specific symptoms. Several explanatory variables were differentially connected to different anxiety disorders (see Table 3). The most important were: (i) child's sleeping difficulties associated with generalized anxiety [Odds Ratio (OR) = 1.19; Confidence Interval (CI) 95% = 1.13–1.25; *p* < .001], social anxiety (OR = 1.07; CI 95% = 1.01–1.13; *p* = .012) and acute stress reaction (OR = 1.09; CI 95% = 1.02–1.17; *p* = .008); (ii) maternal postnatal anxiety associated with generalized anxiety (OR = 1.11; CI 95% = 1.08–1.15; *p* < .001), specific phobia (OR = 1.03; CI 95% = 1.00–1.06; *p* = .022) and acute stress reaction (OR = 1.08; CI 95% = 1.03–1.13; *p* = .001); and (iii) maternal postnatal depression associated with generalized anxiety (OR = 1.04; CI 95% = 1.01–1.06; *p* = .004), specific phobia (OR = 1.03; CI 95% = 1.01–1.05; *p* = .003) and social anxiety (OR = 1.05; CI 95% = 1.03–1.08; *p* < .001).

**TABLE 3:** Associations between risk factors with persistent high levels of anxiety disorders from 8 to 13 years old

	Persistent generalized anxieties (*N* = 6118)			Persistent specific phobia (*N* = 6117)			Persistent social anxiety (*N* = 6117)			Persistent acute stress (*N* = 6117)		
Risk factors	*OR*	*CI 95%*	*p*	*OR*	*CI 95%*	*p*	*OR*	*CI 95%*	*p*	*OR*	*CI 95%*	*p*
Child's sex	0.84	0.69–1.04	.115	**0.38**	**0.32 to 0.44**	**<.001**	0.82	0.67–1.01	.058	0.86	0.66–1.12	.270
Child's birth weight, kg	**0.80**	**0.65 to 0.97**	**.024**	**0.83**	**0.71 to 0.96**	**.013**	0.89	0.73–1.08	.224	1.17	0.91–1.51	.213
Child's sleep difficulties	**1.19**	**1.13 to 1.25**	**<.001**	1.02	0.98–1.06	.335	**1.07**	**1.01 to 1.13**	**.012**	**1.09**	**1.02 to 1.17**	**.008**
Child's ethnicity	0.95	0.46–1.95	.880	0.63	0.38–1.06	.085	0.85	0.42–1.72	.656	**0.42**	**0.21 to 0.85**	**.016**
FAI total score	1.01	0.98–1.03	.670	0.98	0.96–1.00	.128	1.02	0.99–1.04	.200	**1.07**	**1.02 to 1.17**	**<.001**
Maternal age when birth	0.98	0.96–1.01	.154	**1.05**	**1.03 to 1.07**	**<.001**	**1.03**	**1.01 to 1.06**	**.004**	1.00	0.98–1.04	.656
Maternal postnatal depression	**1.04**	**1.01 to 1.06**	**.004**	**1.03**	**1.01 to 1.05**	**.003**	**1.05**	**1.03 to 1.08**	**<.001**	0.99	0.95–1.02	.399
Maternal postnatal anxiety	**1.11**	**1.08 to 1.15**	**<.001**	**1.03**	**1.00 to 1.06**	**.022**	1.01	0.98–1.05	.431	**1.08**	**1.03 to 1.13**	**.001**
Maternal bonding at 8 months	0.99	0.96–1.01	.283	**0.98**	**0.96 to 1.00**	**.029**	0.98	0.95–1.00	.083	0.99	0.96–1.03	.618
Maternal socio‐economic status	1.00	0.99–1.01	.823	1.00	0.99–1.00	.287	1.00	0.99–1.00	.299	1.00	0.99–1.02	.400
Maternal education	**1.40**	**1.11 to 1.77**	**0.004**	0.96	0.80–1.14	0.606	1.00	0.80–1.25	.996	0.87	0.64–1.17	.361

Abbreviations: CI, Confidence Interval; FAI, Total adversity index; OR, Odd Ratio.

Bold values refer to statistically significant results (*p* < .050).

## DISCUSSION

Our study offers novel findings concerning the trajectories of symptoms of specific anxiety disorders and factors associated with persistent anxiety disorders across middle childhood and early adolescence (i.e., 8–13 years). Firstly, distinct anxiety disorders presented different prevalence and patterns over time. Secondly, we detected persistent high levels of anxiety for specific phobia, social anxiety, acute stress reaction and generalized anxiety. Finally, several risk factors were specifically associated with persistent high levels of anxiety disorder‐specific symptoms.

Specific phobia was the most prevalent and severe anxiety disorder for 8‐ and 10‐year‐old children and decreased over time, both for diagnosis and symptomatology. This follows previous evidence, where specific phobia together with separation anxiety were the most prevalent anxiety disorders in children and adolescents (Beesdo‐Baum & Knappe, 2012), with a decreasing pattern over time. This might be due to the fact that as children develop, they are more exposed to their environment and develop skills to cope with such stressful situations (Wadsworth, 2015). Also following previous research, we found that the prevalence of social anxiety diagnoses increased over time (Kashdan & Herbert, 2001), especially from 10 to 13 years, which was supported by increasing total score over time. For instance, social anxiety has its origins mainly in adolescence, a period characterized by shifts in socio‐emotional behaviour and increased vulnerability to social anxiety (Ferri et al., 2014). The differences observed in some anxiety disorders when we compared diagnosis versus symptomatology are important. For instance, separation anxiety was the second most prevalent diagnosis at all time points, while the symptomatology of separation anxiety showed the lowt total scores. Other differences appeared in generalized anxiety, which was the least prevalent anxiety diagnosis at all time points, whilst the symptomatology of generalized anxiety showed high total scores at all time points. This discrepancy between anxiety diagnosis and symptomatology, especially in separation anxiety and generalized anxiety suggests that total scores, which indicate anxiety symptomatology, do not necessarily indicate clinical relevance or provide us with information about level of disability. However, other potential explanation could be that the subscales may differ in how well they predict the presence of the corresponding disorder. Therefore, further research to understand this discrepancy.

Latent class growth analyses detected a group of individuals characterized by persistent high levels of anxiety, specifically in specific phobia, social anxiety, acute stress reaction and generalized anxiety. Patterns of development of anxiety disorders in children and adolescence are an under‐researched area, despite their high prevalence, impact on social and educational functioning (Mikami et al., 2011) and associations with other mental disorders in adulthood (Woodward & Fergusson, 2001). Our main findings suggest that there is a specific group of children and adolescents who are especially prone to persistent and severe anxiety disorders. This highlights the relevance of addressing these disorders as early as possible. In addition, and although this was not the focus of our study, it is important to highlight that anxiety disorders are highly comorbid with each other (Goldstein‐Piekarski et al., 2016), and thus this should be also taken into account when interpreting our findings.

We also examined health‐related and socio‐demographic risk factors related to persistent high levels of anxiety disorder‐specific symptoms. We found that different risk factors distinctively associated with each anxiety disorder. We did not find a common risk factor for all disorders, but we found a tendency for overlap of three specific risk factors which were related to most of the persistent anxiety disorder‐specific symptoms. More specifically, child's sleep difficulties, and postnatal maternal depression and anxiety were associated with 3 out of 4 of the anxiety disorders, suggesting that these were the most relevant health‐related factors. Critically, these are also modifiable (i.e., more treatable) and acting on these could potentially reduce the risk of future persistent and disabling anxiety. Further, such risk factors need to be assessed as these may predict a more prolonged and severe course of illness. Postnatal maternal psychopathology is related to offspring anxiety (Rees et al., 2019), and here we found that both postnatal depression and anxiety lead to persistent anxiety disorders in their offspring across childhood and adolescence. One of the novel findings of this study is the role of early sleep difficulties as risk factors for persistent high levels of anxiety disorders. Sleep disturbances are highly prevalent in anxiety (Staner, 2003). Further, sleep problems in childhood associate with anxiety in adulthood (Gregory et al., 2005). Here we add that childhood sleep problems also relate to persistent high levels of anxiety, across childhood and adolescence. Finally, maternal socio‐economic status was not associated with any of the persistent high levels of anxiety disorder‐specific symptoms detected in our study. Despite the existing evidence suggesting that children and adolescents with low socio‐economic status are at higher risk to develop mental health problems than those with high socio‐economic status (Reiss, 2013), the lack of associations in our study may suggest that socio‐economic status might have an indirect but not direct impact on the development of persistent anxiety; however, further research is required to confirm our findings.

Our study has some limitations. First, there was significant attrition across follow‐up time points. However, we used procedures to ensure the representativeness of our results. Second, our study was unable to utilise data in those older than 13 years, so we cannot comment on anxiety disorders trajectories after this age. Third, other potential risk factors related to general functioning, school performance and interpersonal skills were not explored. Fourth, similar to most epidemiological studies, the anxiety disorders were not clinical diagnosis, but DAWBA‐derived diagnosis. Fifth, we did not test for the fit of non‐linear growth trends, due to challenges around modelling non‐linear growth models with three data points. Sixth, in this study we focused on predictors of persistent 'symptoms' rather than diagnosis of anxiety disorders, and thus future research should also consider potential predictors for diagnosis. Seventh, in this study the only measure to capture anxiety disorders and anxiety symptoms was the DAWBA. Therefore, the prevalence rates for anxiety disorders reported in this study might differ to other studies. Eight, the total scores of each of the DAWBA anxiety dimensions presented slightly different ranges; therefore, this does not allow us to compare across dimensions, but rather we describe each of them separately. Finally, maternal anxiety/depression and severe anxiety in the offspring might be partially due to shared genetic liability to these disorders, which is a factor that we did not account for in this study. Further, using parent‐reported measures for anxiety disorders could lead to underreporting the severity of the problem.

In summary, our findings show that a small group of children and adolescents suffer from frequent and severe anxiety. Further, different risk factors are specifically associated with different anxiety disorders, with child sleeping difficulties and postnatal maternal depression and anxiety being the most relevant. These results are important contribution for future epidemiological studies and clinical studies. Future research is required to elucidate the roles of protective factors and therapeutic involvement across a wider range of age groups to identify those most at risk for enduring and severe anxiety disorders.

## AUTHOR CONTRIBUTIONS

Isabel Morales‐Muñoz, Steven Marwaha, Danielle Hett, Clara Humpston and Pavan K. Mallikarjun, designed the study. Isabel Morales‐Muñoz, Danielle Hett and Clara Humpston were primarily responsible for data analysis and writing of the article. Isabel Morales‐Muñoz, Steven Marwaha, Danielle Hett, Clara Humpston and Pavan K. Mallikarjun, contributed critically to the writing of the article.

## CONFLICTS OF INTEREST

The authors have declared that they have no competing or potential conflicts of interest.

## ETHICS STATEMENT

Ethical approval was obtained from the ALSPAC Law and Ethics Committee and local research ethics committees. Informed consent was obtained from participants following the recommendations of the ALSPAC Ethics and Law Committee.

## Supporting information

Supporting Information 1

## Data Availability

The ALSPAC data used for this study can be provided by ALSPAC pending scientific review and a completed material transfer agreement. Requests for the ALSPAC data should be submitted to: alspac‐data@bristol.ac.uk.
