# Supplementary material for: Anxiety disorders across middle childhood and early adolescence in a UK population‐based cohort
Source: JCPP Adv. 2022 Jun 24;2(3):e12089. doi: 10.1002/jcv2.12089 (PMC10242895; doi:10.1002/jcv2.12089)
Supplement: Supplementary file 1 — Supporting Information 1 [file JCV2-2-e12089-s001.docx]

**Supporting Information**

1. Further details of ALSPAC cohort (page 2)
2. Further details of the DAWBA (page 3)
3. Table S1. Description of the items for each of the DAWBA anxiety total scores (page 4-6)
4. Table S2. Description of subscales/variables for each of the predictors included in the study (pages 7-8)
5. Table S3. Differences in socio-demographic and clinical variables between non-participants and participants (page 9)
6. Table S4. Prevalence of cases per class, for the best model fit of DAWBA anxiety disorders (page 10)
7. Table S5. LCGA model fit values for classes 2-6, for each DAWBA subtypes total scores, separate for boys and girls (page 11)
8. Table S6. Comparisons between persistent classes among all DAWBA-derived Anxiety subtypes (page 12)
9. Figure S1. Growth trajectories of specific phobia and social anxiety across childhood to adolescence, separate for boys and for girls (page 13)
10. Figure S1. Growth trajectories of generalized anxiety across childhood to adolescence, separate for boys and for girls (page 14)

**Further details of ALSPAC cohort:**

The initial number of pregnancies enrolled was 14,541 (for these at least one questionnaire was returned, or a “Children in Focus” clinic had been attended by 19/07/99). Of these initial pregnancies, there was a total of 14,676 foetuses, resulting in 14,062 live births and 13,988 children who were alive at 1 year of age. When the oldest children were approximately 7 years of age, an attempt was made to bolster the initial sample with eligible cases who had failed to join the study originally. As a result, in our study, as some variables were collected from the age of seven onwards there were data available for more than the 14,541 pregnancies mentioned above. Informed consent for the use of data collected via questionnaires and clinics was obtained from participants following the recommendations of the ALSPAC Ethics and Law Committee at the time. Ethical approval was obtained from the ALSPAC Law and Ethics committee and the local research ethics committees.

**Further details of the DAWBA:**

The DAWBA is a package of interviews, questionnaires and rating techniques designed to generate ICD-10 and DSM-IV or DSM-5 psychiatric diagnoses about 2-17 years old. The DAWBA includes a mix of ‘closed’/structured questions and open-ended questions, where respondents describe their difficulties in their own words. The full DAWBA package covers the following diagnoses: Separation anxiety, Specific phobia, Social phobia, Panic disorder/agoraphobia, Post-traumatic stress disorder, Obsessive compulsive disorder, Generalized anxiety disorder, Body dysmorphic disorder, Disruptive mood dysregulation disorder, Major depression, ADHD/hyperkinesis, Oppositional defiant disorder, Conduct disorder, Eating disorders, including anorexia, bulimia and binge eating, Autism spectrum disorders, Tic disorders, including Tourette syndrome, and Bipolar disorders. For each of these disorders, the interview asks about all the symptoms, and other criteria needed for an operationalized diagnosis according to both DSM-IV (American Psychiatric Association, 1994) and the research diagnostic version of ICD-10 (World Health Organisation, 1994). Panic disorder, agoraphobia, autistic disorders, eating disorders, tic disorders, and any other concerns are covered more briefly, with clinical diagnoses of these disorders being correspondingly more dependent on rating the open-ended transcript. The time frame of the interview is the present and the recent past. For many disorders, the ICD-10 and DSM-IV diagnostic criteria stipulate that the symptoms need to have persisted for a specified number of months, e.g. a minimum of 6 months for hyperactivity, oppositional-defiant disorder, and generalized anxiety disorders. In these instances, the relevant section of the DAWBA interview focuses on the child’s symptoms over this stipulated period. The time frame is longest for conduct disorder (since DSM-IV criteria include the number of relevant behaviours displayed over the previous 12 months), and shortest for most of the emotional disorders, where the focus is on the last month, in line with previous recommendations (Shaffer et al., 1996).

**Table S1. Description of the items for each of the DAWBA anxiety total scores**

| **Separation Anxiety score** |
| --- |
| 1. In past month child has often worried about something unpleasant happening to or losing special person. 2. In past month child has often worried about being taken away from special person. 3. In past month child has not wanted to go to school in case something bad happened to special person. 4. In past month child has worried about sleeping alone. 5. In past month child has left bed at night to check on or to sleep near special person. 6. In past month child has worried about sleeping in a strange place. 7. In past month child has been afraid to be alone in room without special person. 8. In past month child has had bad dreams about separation from special person. 9. In past month child has felt ill when had to leave special person. 10. In past month child has become upset at being apart from special person. |
| **Particular Fears score** |
| 1. Child is scared of insects, spiders, wasps, bees, mice, snakes, birds or other creatures. 2. Child is scared of storms, thunder, heights or water. 3. Child is scared of blood, injections or injury. 4. Child is scared of dentists or doctors. 5. Child is scared of other specific situations. 6. Child is scared of the dark. 7. Child has other specific fear. |
| **Social Fears score** |
| 1. In past month child has been afraid of meeting new people. 2. In past month child has been afraid of meeting a lot of people. 3. In past month child has been afraid of speaking in class. 4. In past month child has been afraid of reading out loud in front of others. 5. In past month child has been afraid of writing in front of others. 6. In past month child has been afraid of eating in front of others |
| **Stress Reactions score** |
| 1. In past month child has had vivid memories of event. 2. In past month child has had repeating distressing dreams of event. 3. In past month child has been upset when reminded of event. 4. In past month child has avoided thinking about event. 5. In past month child has avoided activities, places or people that remind of event. 6. In past month child has blocked out important details of event from memory. 7. In past month child has shown less activity in activities. 8. In past month child has expressed smaller range of feelings. 9. In past month child has had problems sleeping. 10. In past month child has seemed irritable or angry. 11. In past month child has had difficulty concentrating. 12. In past month child has always been on the alert for possible danger . 13. In past month child has jumped at small noises. |
| **General Anxieties Score** |
| 1. Child worries about past behavior.      1. Child worries about school-work.      1. Child worries about disasters.      1. Child worries about own health.      1. Child worries about bad things happening to others. 2. Child worries about the future.      1. Child worries about other things. |
| **General Anxieties Symptoms Score** |
| 1. Worries lead to child being restless, tense or on edge. 2. Worries lead to child being easily tired. 3. Worries lead to difficulties in concentrating. 4. Worries lead to irritability.      1. Worries lead to child looking tense.      1. Worries interfere with sleep. |

**Table S2. Description of subscales/variables for each of the predictors included in the study**

| **Family Adversity Index Variables** |
| --- |
| (1) Age of mother at first pregnancy; (2) Housing, comprising (a) adequacy, (b) basic amenities, and (c) defects, damp, and infestation; (3) Mother's and father's low educational attainment; (4) Financial difficulties; (5) Relationship with partner, comprising (a) status, (b) lack of affection, (c) cruelty, and (d) lack of support; (6) Family, comprising (a) size and (b) child in care, not with natural mother, or on at-risk register; (7) Social network, comprising (a) lack of emotional support and (b) lack of practical support; (8) Substance abuse; (9) Crime, comprising (a) being in trouble with the police and (b) convictions; and (10) Psychopathology of the mother (anxiety, depression, or suicide attempts). |
| **Edinburgh Postnatal Depression Scale Items** |
| (1) I have been able to laugh and see the funny side of things; (2) I have looked forward with enjoyment to things; (3) I have blamed myself unnecessarily when things went wrong; (4) I have been anxious or worried for no good reason; (5) I have felt scared or panicky for no good reason; (6) Things have been getting to me; (7) I have been so unhappy that I have had difficulty sleeping; (8) I have felt sad or miserable; (9) I have been so unhappy that I have been crying; (10) The thought of harming myself has occurred to me. |
| **Crown-Crisp Index – Anxiety subscale description** |
| In the ALSPAC cohort, information on mental health status during pregnancy was collected  at around 18 and 32 weeks of pregnancy using a modified questionnaire based on the  Crown Crisp Experiential Index (CCEI) (Crown and Crisp, 1966). This questionnaire had been  reduced from the original 48 items to 23, with responses standardized to four distractors  (“never”, “sometimes”, “often”, “very often”). The CCEI was developed in the mid-1960s and  was divided in a set of six subscales (somatization, depression, free floating anxiety, phobic  anxiety, obsessive compulsive symptoms and hysteria). However, the items in these  subscales do not correspond to the modern diagnostic definitions of the syndromes they are  named after. Moreover, early factor analyses (Alderman et al., 1983) found a substantial  overlap between subscales. A group of experts from the ALSPAC with extensive clinical and epidemiological experience selected five items: “troubled by dizziness or shortness of breath”, “felt as though you may faint”, “feel sick or have indigestion”,” tingling or prickling sensations in  body arms or legs” and “extra sweating” from the CCEI. These items were chosen because of  their similarity with the ICD-10 and DSM-IV definitions of panic disorder and were judged  representative of symptoms of somatic anxiety in mothers (American Psychiatric  Association, 2000). Items were grouped and evaluated as a “somatic anxiety factor” using  confirmatory factor analysis in two different populations (pregnant women and partners of  these women (Bolea-Alamanac and Davies, 2016)). |
| **Maternal bonding score** |
| The maternal enjoyment of baby subscale consists of five items for example, ‘I really enjoy my baby’ and ‘it is a great pleasure to watch my baby develop’. The maternal confidence subscale comprises six items including ‘I feel confident with my baby’ and ‘I feel constantly unsure if I’m doing the right thing for my baby’. Participants rated how applicable the statement was to their personal feelings from 1 = never feel to 4 = exact feeling for each of the items. Overall ‘maternal bonding’ score was obtained from combining the two subscale scores with a range of potential scores going from 4–44. The higher the score the greater maternal bonding with the child. |
| **Cambridge Social Interaction and Stratification Scale description** |
| The CAMSIS measures occupational structure based upon social interactions. Scores can range between 1 (least advantaged) and 99 (most advantaged) with a mean of 50 and a standard deviation of 15 in the population. |
| **Children sleep difficulties total score at 3.5 years, items** |
| (1) CH Refused to go to Bed Past Year; (2) CH Woke Very Early Past Year; (3) CH Has Problem Going To Sleep Past Year; (3) CH Had Nightmares Past Year; (4) CH gets up After Put to Bed Past Year; (5) CH Woke in Night Past Year; (6) CH Gets up After Few HRs Sleep Past Year. |

**Table S3.** Differences in socio-demographic variables between non-participating and participating subjects in the study

|  | **Non-participating group in the study**  **N=7523** | | **Participating group in the study (8 years) N=8122** | | | **Non-participating versus participating** | |
| --- | --- | --- | --- | --- | --- | --- | --- |
|  | *Mean* | *SD* | *Mean* | *SD* | | *OR (95% CI)* | *p* |
| Maternal age when born | 26.63 | 5.13 | 28.96 | 4.60 | | 1.105 (1.097, 1.113) | <0.001 |
| Gestational age | 36.97 | 7.83 | 39.45 | 1.87 | | 1.123 (1.111, 1.135) | <0.001 |
| Birth weight, grams | 3.33 | 0.62 | 3.42 | 0.55 | | 1.322 (1.247, 1.402) | <0.001 |
| Family Adversity score | 5.35 | 4.78 | 4.06 | 4.10 | | 0.937 (0.928, 0.946) | <0.001 |
|  | **Non-participating group in the study** | | **Participating group in the study** | | |  |  |
|  | *N* | *%* | *N* | | *%* |  |  |
| Sex  Male / Female | 3456 / 3341 | 50.8 / 49.2 | 4235 / 4007 | | 51.4 / 48.9 | 0.512 (0.958, 1.090 | 0.512 |
| Ethnicity |  |  |  | |  |  |  |
| White / Other | 4254 / 181 | 95.9 / 4.1 | 7808 / 145 | | 98.2 / 1.8 | 2.291 (1.836, 2.860) | <0.001 |

The individuals associated with attrition in adolescence had younger mothers when the baby was born, shorter gestational age, weighted less at birth, reported higher family adversity problems and were more frequently non-white.

|  | **Class 1** | | **Class 2** | | **Class 3** | |
| --- | --- | --- | --- | --- | --- | --- |
|  | N | % | N | % | N | % |
| **Specific phobia** | 3116 | 20.5 | 11168 | 73.6 | 881 | 5.8 |
| **Social anxiety** | 1841 | 12.1 | 12808 | 84.5 | 516 | 3.4 |
| **Acute stress reaction** | 292 | 1.9 | 14873 | 98.1 | ---- | ---- |
| **Generalized anxieties, composite** | 6331 | 72.9 | 1882 | 21.7 | 469 | 5.4 |

**Table S4.** Prevalence of cases per class, for the best model fit of DAWBA anxiety disorders

**Table S5.** Bayesian Information Criterion, Vuong-Lo-Mendell-Rubin Likelihood Test P Values and Entropy for classes 2-6, for each DAWBA subtypes total scores, separate for boys and girls

|  | **Boys** | | | **Girls** | | |
| --- | --- | --- | --- | --- | --- | --- |
| **Specific phobia** | **BIC** | **VLMR-P** | **Entropy** | **BIC** | **VLMR-P** | **Entropy** |
| 2 classes | 33471.593 | <0.001 | 0.757 | 31847.049 | <0.001 | 0.783 |
| 3 classes | 32976.037 | 0.2167 | 0.749 | 31531.222 | 0.0129 | 0.690 |
| 4 classes | 32791.074 | 0.1570 | 0.729 | 31428.714 | 0.0096 | 0.627 |
| 5 classes | 32670.557 | 0.5077 | 0.644 | 31315.703 | 0.0050 | 0.655 |
| 6 classes | 32530.769 | 0.0522 | 0.622 | 31274.524 | 0.0803 | 0.606 |
| **Social anxiety** |  |  |  |  |  |  |
| 2 classes | 41773.195 | <0.001 | 0.921 | 40155.353 | <0.001 | 0.919 |
| 3 classes | 40626.902 | 0.0401 | 0.893 | 39174.929 | 0.0002 | 0.889 |
| 4 classes | 39815.838 | 0.1113 | 0.893 | 38047.567 | 0.0247 | 0.894 |
| 5 classes | 38645.637 | 0.0041 | 0.913 | 36803.617 | 0.0609 | 0.913 |
| 6 classes | 38671.134 | 0.6438 | 0.646 | 36828.995 | 0.6734 | 0.906 |
| **Acute stress reaction** |  |  |  |  |  |  |
| 2 classes | 17327.145 | 0.0392 | 0.991 | 16465.786 | 0.2659 | 0.986 |
| 3 classes | 17123.520 | 0.0114 | 0.747 | 16302.279 | 0.3244 | 0.779 |
| 4 classes | 13785.952 | 0.0115 | 0.791 | 13530.164 | 0.2809 | 0.826 |
| 5 classes | 12134.977 | 0.6980 | 0.819 | 11002.136 | 0.8608 | 0.989 |
| 6 classes | 10457.734 | 1.0000 | 0.841 | 11027.128 | 0.8062 | 0.878 |
| **Separation anxiety** |  |  |  |  |  |  |
| 2 classes | 39532.991 | 0.1588 | 0.986 | 39669.027 | 0.0049 | 0.972 |
| 3 classes | 37799.053 | 0.0930 | 0.975 | 37791.555 | 0.0584 | 0.951 |
| 4 classes | 36425.664 | 0.7730 | 0.952 | 36493.778 | 0.0927 | 0.939 |
| 5 classes | 34958.311 | 0.4304 | 0.947 | 35850.291 | 0.1718 | 0.929 |
| 6 classes | 34592.758 | 0.4295 | 0.895 | 35381.395 | 0.6265 | 0.643 |
| **Generalized anxiety** |  |  |  |  |  |  |
| 2 classes | 23394.487 | <0.001 | 0.866 | 24087.157 | <0.001 | 0.840 |
| 3 classes | 22452.394 | 0.0138 | 0.879 | 23368.128 | 0.0121 | 0.849 |
| 4 classes | 21649.202 | 0.0542 | 0.893 | 23268.364 | 0.1577 | 0.812 |
| 5 classes | 21085.114 | <0.001 | 0.912 | 22688.971 | 0.7690 | 0.850 |
| 6 classes | 21110.296 | 0.6059 | 0.844 | 22714.044 | 0.9959 | 0.822 |

BIC=Bayesian information criterion; VLMR-P= Vuong-Lo-Mendell-Rubin likelihood ratio test.

*Highlighted in orange color=the class with best model fit in that specific anxiety dimension.

**Table S6.** Comparisons between persistent classes among all DAWBA-derived Anxiety subtypes

|  | Persistent Particular Fears | | | Persistent Social Fears | | | Persistent Stress Reaction | | |
| --- | --- | --- | --- | --- | --- | --- | --- | --- | --- |
|  | X^2^ | P | Persistent levels in both | X^2^ | P | Persistent levels in both | X^2^ | P | Persistent levels in both |
| Persistent GAD | 129.51 | <0.001 | N=84 (17.9%) | 135.81 | <0.001 | N=61 (13%) | 111.75 | <0.001 | N=40 (8.5%) |
|  | Persistent GAD | | | Persistent Social Fears | | | Persistent Stress Reaction | | |
| Persistent Particular Fears | 129.51 | <0.001 | N=84 (17.9%) | 510.72 | <0.001 | N=148 (16.8) | 166.22 | <0.001 | N=68 (7.7%) |
|  | Persistent GAD | | | Persistent Particular Fears | | | Persistent Stress Reaction | | |
| Persistent Social Fears | 135.81 | <0.001 | N=61 (11.8%) | 510.72 | <0.001 | N=148 (16.8) | 170.53 | <0.001 | N=50 (9.7%) |
|  | Persistent GAD | | | Persistent Particular Fears | | | Persistent Social Fears | | |
| Persistent Stress Reaction | 111.75 | <0.001 | N=40 (8.5%) | 166.22 | <0.001 | N=68 (7.7%) | 170.53 | <0.001 | N=50 (9.7%) |


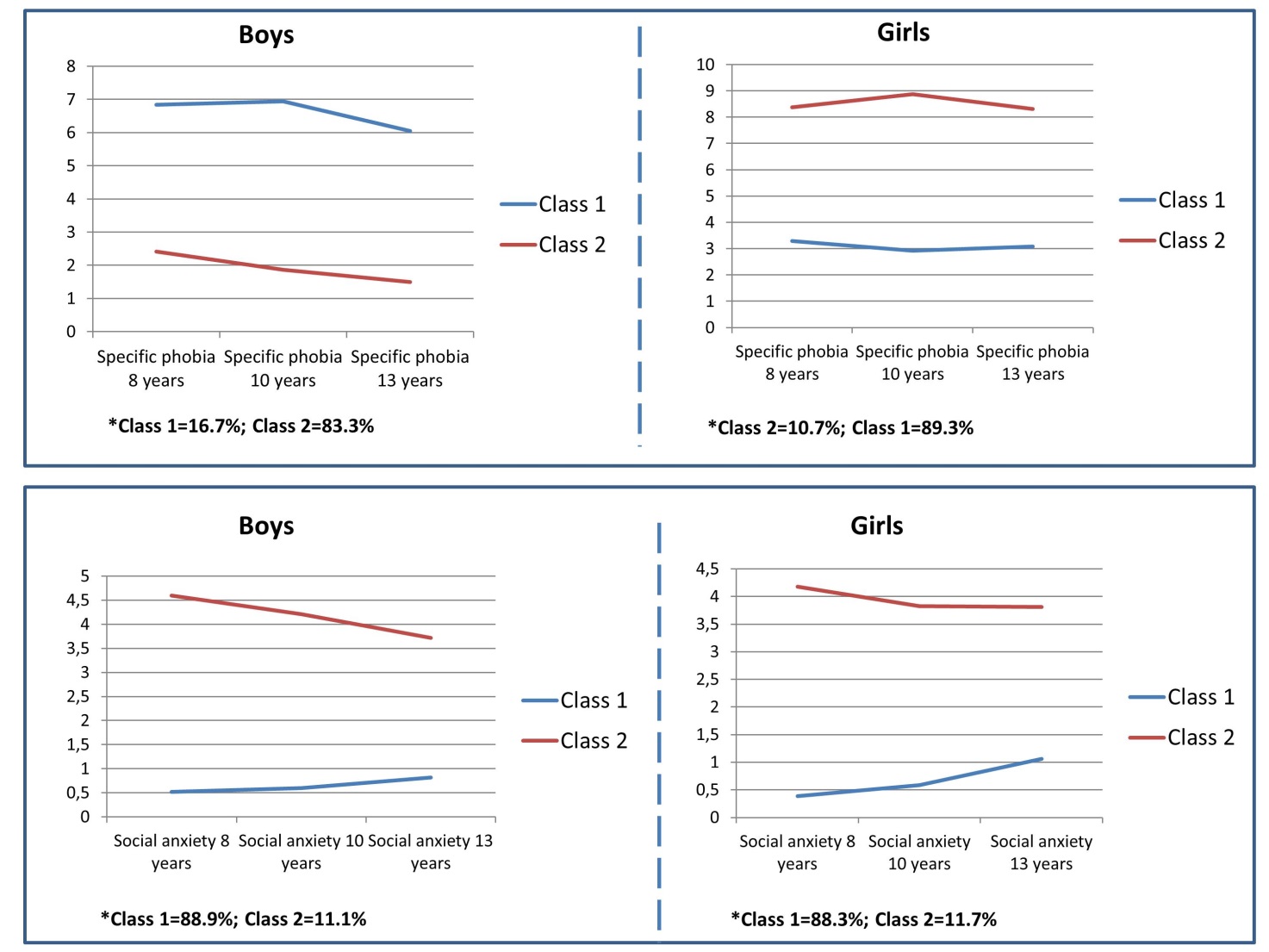


**B**

**A**

**Fig S1a. Growth trajectories of specific phobia and social anxiety across childhood to adolescence, separate for boys and for girls**. The LCGA detected a best model fit for 2 classes for both anxiety disorders and also for boys and girls. **Graph A** shows that for boys, there is a class 1 (blue line) which represents persistent high levels of specific phobia across time points, representing 16.7% of the sample; and a class 2 (red line) which reflects persistent low levels of specific phobia, comprising 83.3% of the sample. For girls, we have a similar pattern, with a class 2 (red line) representing persistent high levels of specific phobia and comprising 10.7% of the sample, and a class 1 (blue line) reflecting persistent low levels of specific phobia and comprising 89.3% of the sample. **Graph B** provided information about the grow trajectories of social anxiety, and similar to specific phobia, similar trend was observed for boys and girls. For boys, we found that class 2 (red line) represented persistent high levels of social anxiety (11.1%) and a class 1 (blue line) representing persistent low levels of social anxiety (88.9%). For girls, we observed also a class 2 (red line) reflecting persistent high levels of social anxiety (11.7%) and a class 1 (blue line) representing persistent low levels of social anxiety (88.3%).

**
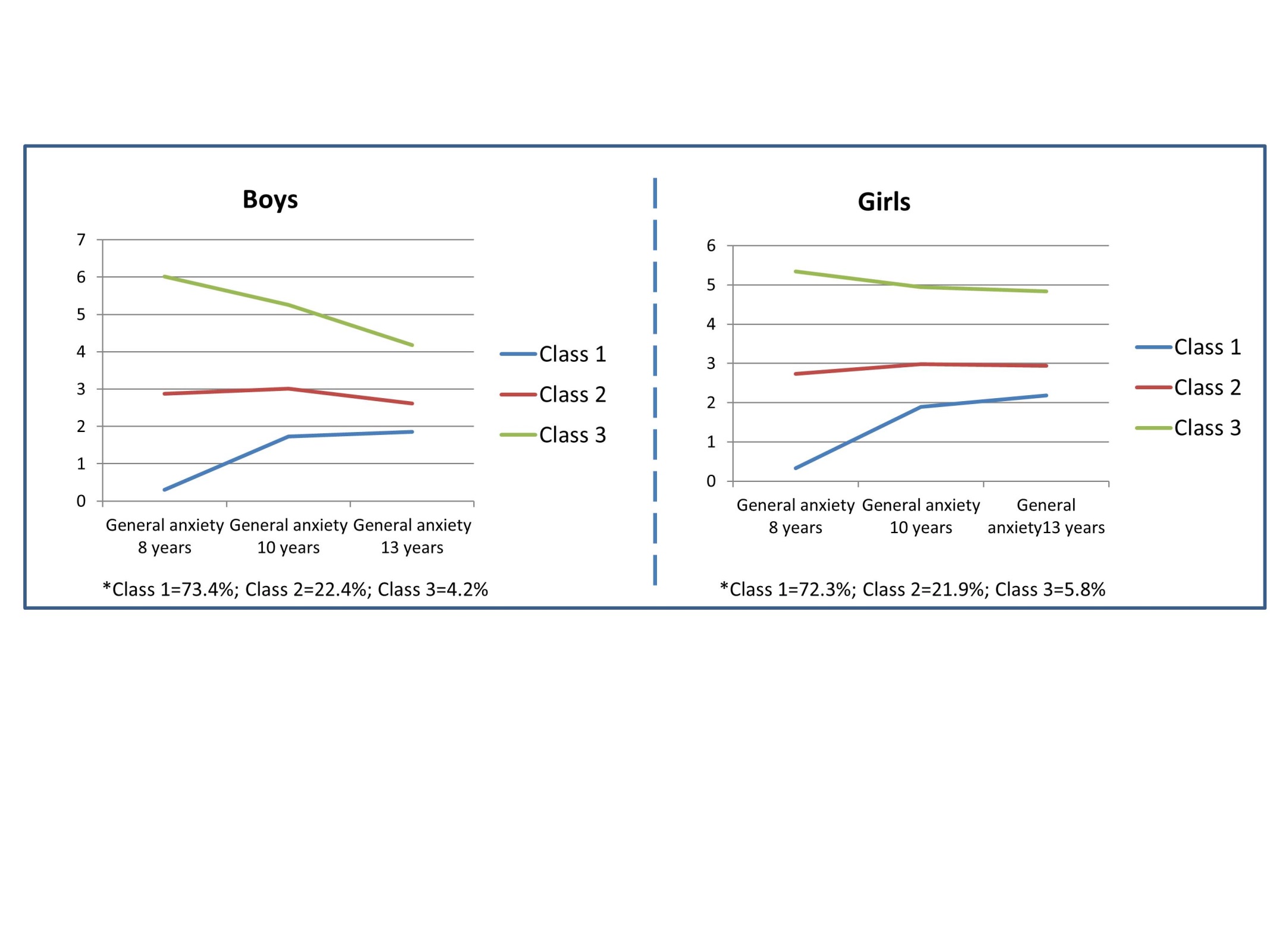
**

**Fig S1b.** **Growth trajectories of generalized anxiety across childhood to adolescence, separate for boys and for girls**. The LCGA detected a best model fit for 3 classes for generalized anxiety, for boys and girls. For boys, we found a class 3 (green line) which represented persistent high levels of generalized anxiety across time points, representing 4.2% of the sample; a class 2 (red line) which reflected persistent moderate levels of generalized anxiety, comprising 73.4% of the sample; and a class 1 (blue line) characterized by persistent low levels of generalized anxiety (73.4%). For girls, we observed a similar pattern, with a class 3 (green line) representing persistent high levels of generalized anxiety across time points (5.8% of the sample); a class 2 (red line) which reflected persistent moderate levels of generalized anxiety, comprising 72.3% of the sample; and a class 1 (blue line) characterized by persistent low levels of generalized anxiety (72.3%).
